# Internet parent–child interaction therapy (I-PCIT) in medically ill child: A case report

**DOI:** 10.1097/MD.0000000000027547

**Published:** 2021-10-15

**Authors:** Valeria Melo, Michael Zaccariello, Emma Girard, Paul Croarkin, Magdalena Romanowicz

**Affiliations:** a Mayo Clinic Alix School of Medicine, Mayo Clinic Rochester, MN; b Department of Psychiatry and Psychology, Mayo Clinic Rochester, MN; c Department of Health Sciences, UC Riverside School of Medicine Riverside, CA.

**Keywords:** attention deficit hyperactivity disorder, medically ill child, parent–child interaction therapy (PCIT), remote-therapy, young children

## Abstract

**Introduction:**

This case illustrates the feasibility, benefit, and putative enhanced ecological validity of performing internet-parent–child interaction therapy (I-PCIT) in the parent–child dyad's home for the treatment of behavior problems in medically ill children in the context of a global pandemic.

**Patient concerns:**

Parents of a 5-year-old girl initially presented with concerns regarding inattentiveness, physical and verbal fighting with her siblings, and getting kicked out of daycare for hitting another child. Patient also had difficulties sleeping at night.

**Diagnoses:**

Patient was diagnosed with electrical status epilepticus in sleep, frontal lobe executive function deficit, and attention deficit hyperactivity disorder.

**Interventions:**

Patient received a course of I-PCIT. Equipment included a cell phone with video capabilities connected to a videotelephony software program and set-up in the child's home by the parents. The treatment course included 8, 1-hour, weekly teaching/coaching sessions (7 of which were performed using I-PCIT) plus 1 follow-up booster session 6 months later.

**Outcomes:**

Home-based I-PCIT implementation greatly improved disruptive behaviors in a young child with electrical status epilepticus in sleep and attention deficit hyperactivity disorder.

**Conclusion:**

A combination of I-PCIT and methylphenidate allowed her to be successful at home and in a school setting. More research is needed on PCIT adaptations, such as home-based and internet-based PCIT, for medically ill children as well as treatment protocols for combined therapies.

**Key point**

- Despite there being a well-documented adverse impact of childhood chronic illness on the functioning of the child and parent–child relationship, there is limited data on parenting interventions for treatment of disruptive behaviors in chronically ill children.
- The current COVID-19 pandemic has exacerbated both the need for treatment of behavior problems in medically ill children and the barriers to receiving the otherwise standard in-person PCIT.
- PCIT adapted for home-based and internet-based sessions successfully reduced disruptive behaviors in a medically ill child.
- Technology needed was minimal: a cell phone with video capabilities and a videotelephony software program.

## 1 Introduction

The prevalence of chronic health conditions in children has progressively increased since the 1960s.^[1]^ The latest epidemiological studies suggest that up to 25% of U.S. children aged 2 to 8 years have chronic health conditions, such as obesity, asthma, other physical conditions, or behavior/learning problems, that interfere with daily activities of life or require medication or specialized health services.^[2]^ The impact of childhood chronic illness can be substantial, affecting both the child and the family unit.

Children with chronic health conditions have an increased risk of impaired social functioning,^[3–4]^ school absenteeism,^[5]^ and lower self-esteem.^[6]^ They are up to 3 times more likely to experience co-morbid emotional, development, and behavioral problems^[7]^ like depression or anxiety (internalizing, emotional problems), difficulty learning, understanding, paying attention, communicating, speaking, or being understood (developmental problems), and aggression, acting-out, fighting, bullying, or arguing (externalizing, behavioral problems).^[3,7,8]^

Psychosocial interventions are considered first line treatment for behavioral problems in younger children, with the most evidence to support behavioral parent training (BPT).^[9–13]^ However, despite the well documented impact of chronic illness on functioning of the child and parent–child relationship, there is limited data on parenting interventions for chronically ill children.^[14,15]^

Parent–child interaction therapy (PCIT) is 1 example of a widely used and evidence-based BPT approach for the treatment of emotional and disruptive behavior disorders in children aged 2 to 7 years.^[16,17]^ Traditional PCIT was developed as a clinic-based program for the treatment of disruptive, externalizing behavior disorders in children aged 3 to 6 years old.^[18]^ A limited number of prior case reports^[19–23]^ and 1 case series^[24]^ have provided support for the application of PCIT in chronically ill or medically complex children. These reports outlined treatment courses where PCIT was either performed within outpatient mental health settings (the setting that PCIT was originally designed for) or adapted to the hospital bedside.

PCIT integrates operant conditioning techniques and play therapy to encourage a secure caregiver–child relationship through 2 phases of treatment: child-directed interaction (CDI) and parent-directed interaction (PDI).^[17]^

The Dyadic parent–child interaction coding system is a well-studied, standardized coding system that is used to assess caregiver's skill goal criteria and determine when it is time to progress from CDI to PDI.^[25,26]^ The Eyberg Child Behavior Inventory is an empirically supported parent report instrument that uses 2 scales to assess frequency (Intensity Scale) and type of behavior problems (Problem Scale) and is also used as PCIT completion criteria, with the goal being to fall within ½ standard deviation of the normative mean and for parents to express self-assurance in handling their child's behavior.^[27–29]^

The current study presents an adaptation of PCIT for telemedicine for the treatment of disruptive behavior problems in the case of a 5-year-old girl with a history of electrical status epilepticus in sleep (ESES), frontal lobe executive function deficit, and attention deficit hyperactivity disorder (ADHD).

## 2 Case presentation

The patient was a 5-year-old Caucasian female with no psychiatric history. She was born full-term via vaginal delivery. Pregnancy, labor, and delivery were uncomplicated, with the exception of a brief period of hypotonia and cyanosis a few minutes after delivery that resolved without need for intubation or oxygenation. There were no concerns for maternal use of alcohol, tobacco, illicit drugs, or prescription medications during the pregnancy. Her developmental milestones were met within the expected timeframes. Family psychiatric history was notable for ADD/ADHD in her 19-year-old sister, autism and ADD/ADHD in her 16-year-old brother, depression in her mother, epilepsy in the patient's maternal aunt and great, great grandfather, and maternal and paternal family history of intellectual disability. Recent family stressors included financial difficulties, insurance coverage difficulties, and extensive traveling related to the passing away of the maternal grandmother. Initial behavioral concerns included inattentiveness, physical and verbal fighting with her siblings, and getting kicked out of daycare for hitting another child.

In October of 2018, approximately a year and a half leading up to the referral to psychiatry for disruptive behavior, the patient underwent an extensive medical work-up that began due to concerns with snoring in the context of behavioral outbursts (hitting and hair pulling), hyperactivity, tonsillar hypertrophy, and severe pediatric obesity (BMI ≥ 99^th^ percentile). An overnight polysomnography to evaluate for sleep disordered breathing incidentally revealed a spike and wave electroencephalogram abnormality that lead to a referral to pediatric neurology for further evaluation. A referral to ear, nose, and throat was also made given the mild upper airway obstruction and an adenotonsillectomy was eventually performed.

Evaluation in the pediatric epilepsy monitoring unit showed persistent spike and slow wave activity in the bifrontal and bioccipital regions consistent with ESES – a rare pediatric epilepsy syndrome that puts her at risk for seizures and global cognitive regression that can manifest primarily as language and behavioral difficulties.^[30,31]^ An MRI was performed which demonstrated no underlying brain abnormalities or evidence of epileptogenic foci. Baseline neuropsychological testing revealed varying levels of provider-observed inattention, impulsivity, distractibility, restlessness, and careless mistake making. Similarly, maternal reporting varied, at times indicating high levels of hyperactivity and aggression and at other times denoting developmentally appropriate behavioral and emotional functioning. Pre-academic skills assessed by the provider fell in a borderline range, indicating moderate impairment. Cognition, in terms of intelligence, language, spatial skills, speed of information processing, and learning new information, was well developed. Mild speech articulation difficulties were noted, as well as some struggles with fine motor coordination. No formal diagnosis was made at this time, but parent management training was discussed as a treatment option in the case of worsening behavioral problems.

The negative MRI was followed up with a referral to medical genetics where a chromosomal microarray and epilepsy gene panel were recommended to search for genetic etiologies of ESES. The patient ultimately had whole exome sequencing done, which found variants of uncertain significance in the GNRIN2A and CHD2 genes. GRIN2A variants, specifically, have been reported in some childhood electroclinical syndromes like continuous spike and wave in slow-wave sleep and Landau–Kleffner syndrome that are associated with ESES.^[32]^ The recommendation at this time was to hold off on antiepileptic medication given the absence of clinical seizures or developmental regression.

The ear, nose, and throat specialist initially assessed the behavioral problems to be daytime manifestations of sleep disordered breathing. However, while the snoring and other obstructive airway symptoms improved after the adenotonsillectomy, the behavioral problems persisted.

With the behavioral concerns progressing, a trial of acetazolamide was initiated. However, despite a significant improvement on acetazolamide in terms of absence of epileptiform discharges recorded on electroencephalogram, behavioral concerns persisted.

Repeat neuropsychological testing done a year a part included suspected depression due to frequent mood changes and irritability, as well as maternal concerns for extreme struggles in the patient's ability to guide and manage behavior and emotions at an age-appropriate level. Teachers indicated struggles with turn taking, instigating bullying, aggression, and unusual behaviors (eg, unaware of surroundings) and social skills.

Taken together, the patient was diagnosed with frontal lobe and executive function deficit at this time. The findings at this time also suggested a diagnosis of ADHD. Although the exact etiology of the behavioral problems or cause for progression of severity remained unclear, parent management training (specifically PCIT) was again discussed and recommended as the first line treatment to target the behavioral concerns before trialing medication.

### 2.1 Intervention

**Figure 1:**
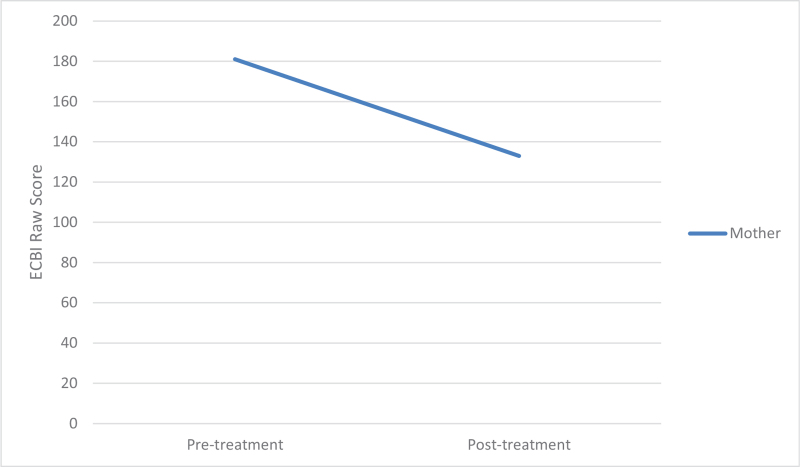
Results for externalizing behavior problems (parent report). ECBI clinical cut-off score is 132. ECBI = Eyberg Child Behavior Inventory.

The patient presented to the clinic with her mother, father, and sister in March of 2020 for an in-person pretreatment assessment of child and family functioning. They were deemed to benefit from PCIT and started psychotherapy. However, after the first CDI teaching session, due to the COVID-19 pandemic, a decision was made to transition to home-based internet-PCIT (I-PCIT) using nothing more than a cell phone with video capabilities that was connected to a videotelephony software program and set-up in the child's home by the parents. During treatment, the mother's ratings of the patient's behavior were tracked at the beginning and at the end of the treatment using the Eyberg Child Behavior Inventory Scale (see Fig. 1). With the sudden closure of schools in Minnesota the family continued to report increased challenges with everyone being at home. The parents reported difficulty with completing the 5 minutes per day of special play time (ie, time spent practicing CDI/PDI skills) due to being busy setting up everyone's online schooling. A considerable time was spent discussing ways the family might organize their schedule and helping the family problem solve around the new challenges with their new daily routine. A scheduled 5 minutes was found every evening before dinner to practice special play time. On session 5 of CDI coaching the parents reported daily practice of special play time and effectiveness of selective ignoring in significantly reducing disruptive behaviors.

### 2.2 Outcome

**Figure 2:**
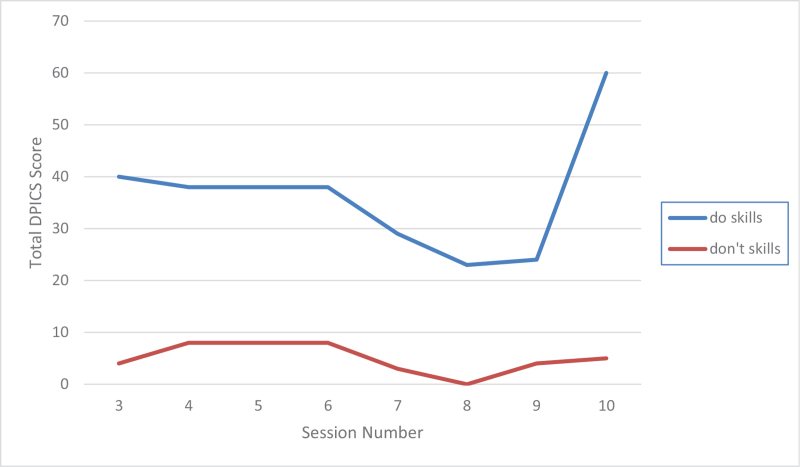
Results of mother's parenting skills (observational) based on DPICS coding during first 5 minutes of appointments. “do skills” are behavior descriptions, reflections, labeled praises; “don’t skills” are questions, commands, and negative talk. DPICS = Dyadic parent–child interaction coding system.

**Figure 3:**
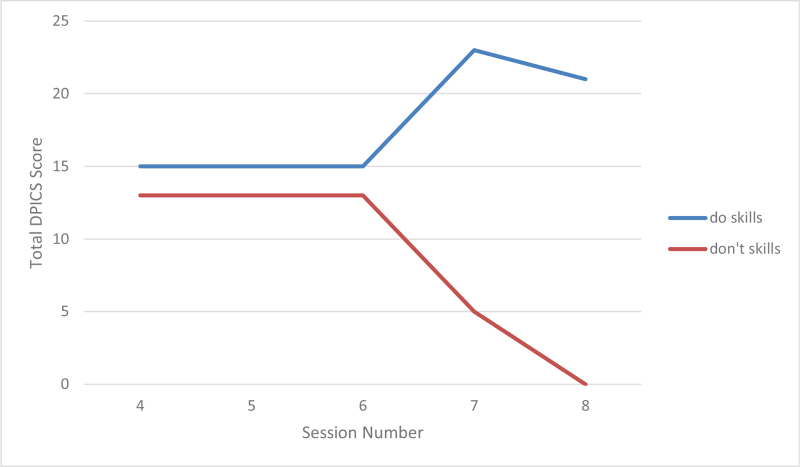
Results of father's parenting skills (observational) based on DPICS coding during first 5 minutes of appointments. “do skills” are behavior descriptions, reflections, labeled praises; “don’t skills” are questions, commands, and negative talk. DPICS = Dyadic parent–child interaction coding system.

**Figure 4:**
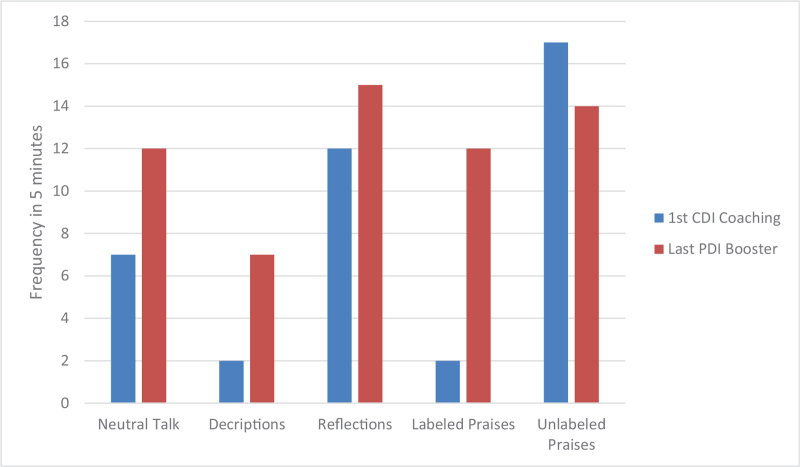
Parent “Do skills” that give positive attention.

**Figure 5:**
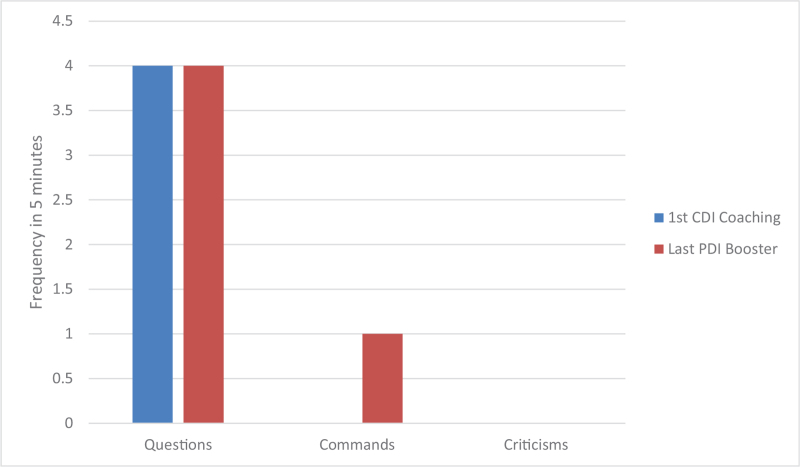
Parent “Don’t skills” that lead to conflict.

Both parents made a tremendous progress in their skills as measured by Dyadic parent–child interaction coding system and illustrated by Figures 2 and 3. During practice of the PDI skills, the patient initially had a difficult time staying in her time-out chair, partially because it was placed in the kitchen with some toys around her. Later she was mostly compliant with parents reporting 2 to 3 time-outs per week. In the sessions the mother had to use occasional time-out warnings but no time-out procedures. Parents described the patient as happy, less impulsive, and more compliant. She continued to struggle with some hyperactive and inattentive behaviors, but they were much more manageable per the parents’ report. Scheduled home-based I-PCIT coaching sessions stopped after a total of 8, 1-hour, weekly sessions. The patient presented for a booster session 6 months later after she was started on methylphenidate by her pediatrician for management of ADHD symptoms (persistent high energy and significant need for redirection in in-person kindergarten leading to difficulty completing assignments). No side effects to the medication were reported at this time. After 2 booster sessions mother was confident in managing patient's defiant behaviors. The parents maintained their Do and Don’t skills as illustrated by Figures 4 and 5. The mother was reportedly very pleased with how well the patient was doing overall in her in-person kindergarten. She was able to follow her academic instruction and work on her small homework assignments after school. The summary of patient's clinical features and treatment is provided in Table 1.

**Table 1:** Summarized clinical features and treatment outcome of the presented case.

	Patient
Age/gender	5/F
Clinical presentation	Inattentiveness, physical and verbal fighting with her siblings, hitting
Co-morbid illnesses	electrical status epilepticus in sleep (ESES), variants of uncertain significance in the GNRIN2A and CHD2 genes found, obstructive sleep apnea
Initial treatments	Adenotonsillectomy, acetazolamide
Treatment for behavioral concerns	Internet-parent–child interaction therapy (I-PCIT)
Outcome	Difficult behaviors decreased to nonclinical levels as measured by Eyberg Child Behavior Inventory (ECBI)

## 3 Discussion

To our knowledge, this is the first case report of a child with a neurologic disorder (ESES) that is associated with neurobehavioral symptomatology and externalizing problems for whom home-based I-PCIT was an effective treatment. There are several case studies of children with medical conditions where externalizing behavior problems improved with standard in-person PCIT.^[19–21,23]^ Our patient started with standard PCIT protocol but due to the COVID-19 pandemic had to be transitioned to I-PCIT after 1 in-person session. A small subset of controlled trials have shown the efficacy of telepsychiatry for providing individual and group based caregiver behavioral training for the treatment of disruptive behaviors and related disorders in children.^[33–36]^ However, only one of these studies looked at fully remote and home-based PCIT specifically^[33]^ and none included children with other significant medical conditions. That being said in a case series by Christian-Brandt and Santacrose (2020)^[24]^ where they discuss various adaptations to bedside PCIT for medically ill children, use of telemedicine is listed as one of the strategies.

Together, these studies have set the stage for the use of telemedicine to address gaps in mental health care for children that are related to barriers like work force shortages and difficulties with cost, transportation, and stigma associated with visiting a mental health facility.^[37–41]^ In light of the well-studied barriers to treatment, in-person home-based PCIT adaptations have been implemented and shown to be comparable in efficacy to clinic-based PCIT interventions.^[42,43]^ While a degree of control is inherently lost, advantages to home-based PCIT include lower attrition rates, enhanced ecological validity in relation to observing “real-life” behaviors in their “natural environment”, increased disruptive behavior identification, and an increased ability to individualize PCIT to families’ actual needs.^[42,44]^ However, problems of treatment availability, accessibility, and acceptability may persist. Home-based I-PCIT, as presented in this case report, is in a unique position to build on the benefits of in-person home-based PCIT and address enduring barriers to care.

This past year, the wider dissemination of telehealth services has been expedited due to the current COVID-19 public health crisis and stay at home orders that have resulted in decreased restrictions and a historic expansion of telehealth insurance coverage.^[45]^ Effective implementation of telepsychiatry is especially imperative during this time, as a rise in mental health problems is expected in relation to quarantine, isolation, and times of increased stress due to the pandemic.^[46]^ These factors outline the timeliness and importance of adapting PCIT to telemedicine to support families that have children with chronic illnesses who are already at increased risk of emotional, development, and behavioral problems, in addition to being immunocompromised or having difficulty leaving home. With the use of nothing more than a cell phone with video capabilities that was connected to a videotelephony software program and set-up in the child's home, this case report demonstrates the feasibility, benefit, and putative enhanced ecological validity of performing I-PCIT for the treatment of behavior problems in medically ill children in the context of a global pandemic. Some of the expected challenges of home-based PCIT did occur, such as additional physical distractions during the sessions (eg, other family members). However, this was not a significant barrier to treatment and likely helped to bridge the gap between efficacy and effectiveness of PCIT in experimental vs real-world settings.

Six months after the PCIT treatment was completed our patient was started on a stimulant medication by her pediatrician to address her symptoms of inattention and hyperactivity which raises interesting questions regarding treatment guidelines. Currently American Academy of Pediatrics (Wolraich et al, 2019^[12]^) recommend that preschool-aged patients (age 4–6 years) with ADHD should undergo evidence-based BPT in behavior management (ie, PCIT) and/or behavioral classroom interventions as the first line of treatment. Medicine such as methylphenidate may be considered if psychotherapy is not successful after at least 8 weeks of treatment. In the present case I-PCIT helped significantly with disruptive behaviors and noncompliance but the patient continued to struggle with a degree of hyperactive and impulsive symptomatology that was disruptive when she entered in-person school. Methylphenidate seemed to help with that. Currently data is lacking on children with ADHD and disruptive behaviors who might benefit from combined treatment.

Our case report had a number of strengths and limitations. The biggest limitation is that it involves a narrow scope which limits its generalizability. The main strengths of our case are that it is comprehensive and well-researched and include a child with a neurologic and psychiatric disorder for whom treatments are very limited and it might be a great reference in the literature for clinicians searching for innovative treatments.

## 4 Conclusion

I-PCIT implementation greatly improved disruptive behaviors in a young child with ESES and ADHD. A combination of I-PCIT and methylphenidate allowed her to be successful at home and in a school setting. More research is needed on PCIT adaptations, such as home-based and internet-based PCIT, for medically ill children as well as treatment protocols for combined therapies.

## Author contributions

**Conceptualization:** Valeria Melo, Michael Zaccariello, Magdalena Romanowicz.

**Project administration:** Valeria Melo.

**Writing – original draft:** Valeria Melo, Michael Zaccariello, Emma Girard, Magdalena Romanowicz.

**Writing – review & editing:** Valeria Melo, Michael Zaccariello, Emma Girard, Paul Croarkin, Magdalena Romanowicz.

## References

[1] Perrin JM Anderson LE Van Cleave J. The rise in chronic conditions among infants, children, and youth can be met with continued health system innovations. Health Aff (Millwood) 2014;33:2099–105. 25489027 10.1377/hlthaff.2014.0832

[2] Van Cleave J Gortmaker SL Perrin JM. Dynamics of obesity and chronic health conditions among children and youth. JAMA 2010;303:623–30. 20159870 10.1001/jama.2010.104

[3] Hysing M Elgen I Gillberg C. Emotional and behavioural problems in subgroups of children with chronic illness: results from a large-scale population study. Child Care Health Dev 2009;35:527–33. 19323670 10.1111/j.1365-2214.2009.00967.x

[4] Meijer SA Sinnema G Bijstra JO. Social functioning in children with a chronic illness. J Child Psychol Psychiatry 2000;41:309–17. 10784078

[5] Eksi A Molzan J Savasir I. Psychological adjustment of children with mild and moderately severe asthma. Eur Child Adolesc Psychiatry 1996;4:77–84. 10.1007/BF01977735 7796253

[6] Sultana S Oommen A Shanmugham V. Psychological adjustment in juvenile diabetics. J Indian Acad Appl Psychol 2007;33:39–46.

[7] Blackman JA Gurka MJ Gurka KK. Emotional, developmental and behavioural co-morbidities of children with chronic health conditions. J Paediatr Child Health 2011;47:742–7. 21449905 10.1111/j.1440-1754.2011.02044.x

[8] Pinquart M Shen Y. Behavior problems in children and adolescents with chronic physical illness: a meta-analysis. J Pediatr Psychol 2011;36:1003–16. 21810623 10.1093/jpepsy/jsr042

[9] Eyberg SM Nelson MM Boggs SR. Evidence-based psychosocial treatments for children and adolescents with disruptive behavior. J Clin Child Adolesc Psychol 2008;37:215–37. 18444059 10.1080/15374410701820117

[10] Comer JS Chow C Chan PT. Psychosocial treatment efficacy for disruptive behavior problems in very young children: a meta-analytic examination. J Am Acad Child Adolesc Psychiatry 2013;52:26–36. 23265631 10.1016/j.jaac.2012.10.001 PMC4247988

[11] Gleason MM Egger HL Emslie GJ. Psychopharmacological treatment for very young children: contexts and guidelines. J Am Acad Child Adolesc Psychiatry 2007;46:1532–72. 18030077 10.1097/chi.0b013e3181570d9e

[12] Wolraich ML Hagan JF Allan C. Subcommittee on children and adolescents with attention-deficit/hyperactive disorder: clinical practice guideline for the diagnosis, evaluation, and treatment of attention deficit/hyperactivity disorder in children and adolescents. Pediatrics 2019;144:e20192528. 31570648 10.1542/peds.2019-2528 PMC7067282

[13] Wyatt Kaminski J Valle LA Filene JH. A meta-analytic review of components associated with parent training program effectiveness. J Abnorm Child Psychol 2008;36:567–89. 18205039 10.1007/s10802-007-9201-9

[14] Durkin K Han RC McNeil CB. Parent–child interaction therapy for children in medical settings. Mental Health 2019;15:878–83.

[15] Morawska A Calam R Fraser J. Parenting interventions for childhood chronic illness: a review and recommendations for intervention design and delivery. J Child Health Care 2015;19:05–17. 10.1177/1367493513496664 24486817

[16] Zisser A Eyberg SM. Kazdin AE Weisz JR. Treating Oppositional Behavior in Children Using Parent– Child Interaction Therapy; in Evidence-Based Psychotherapies for Children and Adolescents. Guilford Press, XXXX. New York, NY:2010.

[17] Lieneman CC Brabson LA Highlander A. Parent–child interaction therapy: current perspectives. Psychol Res Behav Manage 2017;10:239–56. 10.2147/PRBM.S91200 PMC5530857 28790873

[18] McNeil CB Hembree-Kigin TL. Roberts MC. Overview of Parent–Child Interaction Therapy; in Issues in Clinical Child Psychology: Parent–Child Interaction Therapy. Springer, XXXX. Boston, MA:2010.

[19] Bagner DM Fernandez MA Eyberg SM. Parent–child interaction therapy and chronic illness: a case study. J Clin Psychol Med Sett 2004;11:01–6.

[20] Cohen ML Heaton SC Ginn N. Parent–child interaction therapy as a family-oriented approach to behavioral management following pediatric traumatic brain injury: a case report. J Pediatr Psychol 2012;37:251–61. 22004884 10.1093/jpepsy/jsr086

[21] Garcia D Lorenzo N Kuluz J. Parent–child interaction therapy and moderate pediatric traumatic brain injury: a case study. Evid Based Pract Child Adolesc Ment Health 2016. 2379–4933.

[22] Miller EM Eyberg SM. Boggs SR Rodriguez CM. Parent–Child Interaction Therapy with a Diabetic Child; in Advances in Child Health Psychology: Abstracts. Clinical and Health Psychology Publishing, XXXX. Gainesville, FL:1991.

[23] Shafi RMA Vande Voort JL Croarkin PE. Parent–child interaction therapy in a case of global developmental delay and leukoencephalopathy. Front Psychiatry 2018;9:427. 30258371 10.3389/fpsyt.2018.00427 PMC6143813

[24] Christian-Brandt AS Santacrose D. Adapting PCIT to address mental health care disparities among underserved families impacted by pediatric illness: a case series of bedside PCIT. Clin Pract Pediatr Psychol 2020;8:164–75.

[25] Eyberg SM Nelson MM Duke M. Manual for The Dyadic Parent–Child Interaction Coding System. 3rd ed. Thousand Oaks, CA: Sage Publications; 2004.

[26] Eyberg SM Nelson MM Ginn NC. Dyadic Parent–Child Interaction Coding System: Comprehensive Manual for Research and Training. 4th ed. Gainesville, FL: PCIT International; 2013.

[27] Eyberg SM Pincus D. Eyberg Child Behavior Inventory and Sutter–Eyberg Student Behavior Inventory: Professional Manual. Odessa, FL: Psychological Assessment Resources; 1999.

[28] Funderburk BW Eyberg SM Rich BA. Further psychometric evaluation of the Eyberg and Behar rating scales for parents and teachers of preschoolers. Early Educ Dev 2003;14:67–80.

[29] Rich BA Eyberg SM. Accuracy of assessment: the discriminative and predictive power of the Eyberg child behavior inventory. Ambul Child Health 2001;7:249–57.

[30] Nickels K Wirrell E. Electrical status epilepticus in sleep. Semin Pediatr Neurol 2008;15:50–60. 18555191 10.1016/j.spen.2008.03.002

[31] Sánchez Fernández I Loddenkemper T Peters JM. Electrical status epilepticus in sleep: clinical presentation and pathophysiology. J Pediatr Neurol 2012;47:390–410. 10.1016/j.pediatrneurol.2012.06.016 23127259

[32] Myers KA Scheffer IE. Adam MP Ardinger HH Pagon RA. GRIN2A-related speech disorders and epilepsy. XXXX. Seattle, WA: University of Washington; 2016;GeneReviews® [Internet].

[33] Comer JS Furr JM Miguel EM. Remotely delivering real-time parent training to the home: an initial randomized trial of internet-delivered parent–child interaction therapy (I-PCIT). J Consult Clin Psychol 2017;85:909–17. 28650194 10.1037/ccp0000230

[34] Kirkman JJL Hawes DJ Dadds MR. An open trial for an e-health treatment for child behavior disorders II: outcomes and clinical implications. Evid Based Pract Child Adolesc Mental Health 2016;1:213–29.

[35] Tse YJ McCarty CA Vander Stoep A. Teletherapy delivery of caregiver behavior training for children with attention-deficit hyperactivity disorder. Telemed J E Health 2015;21:451–8. 25719609 10.1089/tmj.2014.0132 PMC4458734

[36] Xie J Dixon JF Yee OM. A study on the effectiveness of videoconferencing on teaching parent training skills to parents of children with ADHD. Telemed J E Health 2013;19:192–9. 23405952 10.1089/tmj.2012.0108

[37] Comer JS Barlow DH. The occasional case against broad dissemination and implementation: retaining a role for specialty care in the delivery of psychological treatments. Am Psychol 2014;69:01–18. 10.1037/a0033582 PMC4260460 23915401

[38] Comer JS Furr JM Cooper-Vince C. Rationale and considerations for the internet-based delivery of parent–child interaction therapy. Cogn Behav Pract 2015;22:302–16. 26120268 10.1016/j.cbpra.2014.07.003 PMC4480784

[39] Koerting J Smith E Knowles MM. Barriers to, and facilitators of, parenting programmes for childhood behaviour problems: a qualitative synthesis of studies of parents’ and professionals’ perceptions. Eur Child Adolesc Psychiatry 2013;22:653–70. 23564207 10.1007/s00787-013-0401-2 PMC3826057

[40] Lanier P Kohl PL Benz J. Parent–child interaction therapy in a community setting: examining outcomes, attrition, and treatment setting. Res Soc Work Pract 2011;21:689–98. 10.1177/1049731511406551 PMC4021486 24839378

[41] Owens PL Hoagwood K Horwitz SM. Barriers to children's mental health services. J Am Acad Child Adolesc Psychiatry 2002;41:731–8. 12049448 10.1097/00004583-200206000-00013

[42] Fowles TR Masse JJ McGoron L. Home-based vs. clinic-based parent–child interaction therapy: comparative effectiveness in the context of dissemination and implementation. J Child Fam Stud 2018;27:1115–29.

[43] Ware LM McNeil CB Masse J. Efficacy of in-home parent–child interaction therapy. Child Fam Behav Therapy 2008;30:99–126.

[44] McNeil CB Hembree-Kigin TL. Roberts MC. Home-Based PCIT: From The Lab to The Living Room; in Issues in Clinical Child Psychology: Parent–Child Interaction Therapy. Springer, XXXX. Boston, MA:2010.

[45] Secretary Azar announces historic expansion of telehealth access to combat COVID-19. U.S. Department of Health and Human Services, 2020. Available at: https://www.hhs.gov/about/news/2020/03/17/secretaryazar-announces-historic-expansion-of-telehealth-access-to-combatcovid-19.html. Accessed November 20, 2020.

[46] Gurwitch RH Salem H Nelson MM. Leveraging parent–child interaction therapy and telehealth capacities to address the unique needs of young children during the COVID-19 public health crisis. Psychol Trauma 2020;12:S82–4. 32538646 10.1037/tra0000863

